# Access to Care and Cardiovascular Disease Prevention: A Cross-Sectional Study in 2 Latino Communities

**DOI:** 10.1097/MD.0000000000001441

**Published:** 2015-08-28

**Authors:** Héctor E. Alcalá, Stephanie L. Albert, Dylan H. Roby, Jacob Beckerman, Philippe Champagne, Ron Brookmeyer, Michael L. Prelip, Deborah C. Glik, Moira Inkelas, Rosa-Elenna Garcia, Alexander N. Ortega

**Affiliations:** From the UCLA Fielding School of Public Health, Los Angeles, CA.

## Abstract

Cardiovascular disease (CVD) is the leading killer of Americans. CVD is understudied among Latinos, who have high levels of CVD risk factors. This study aimed to determine whether access to health care (ie, insurance status and having a usual source of care) is associated with 4 CVD prevention factors (ie, health care utilization, CVD screening, information received from health care providers, and lifestyle factors) among Latino adults and to evaluate whether the associations depended on CVD clinical risk/disease.

Data were collected as part of a community-engaged food environment intervention study in East Los Angeles and Boyle Heights, CA. Logistic regressions were fitted with insurance status and usual source of care as predictors of the 4 CVD prevention factors while controlling for demographics. Analyses were repeated with interactions between self-reported CVD clinical risk/disease and access to care measures.

Access to health care significantly increased the odds of CVD prevention. Having a usual source of care was associated with all factors of prevention, whereas being insured was only associated with some factors of prevention. CVD clinical risk/disease did not moderate any associations.

Although efforts to reduce CVD risk among Latinos through the Affordable Care Act could be impactful, they might have limited impact in curbing CVD among Latinos, via the law's expansion of insurance coverage. CVD prevention efforts must expand beyond the provision of insurance to effectively lower CVD rates.

## INTRODUCTION

Cardiovascular disease (CVD) is the leading killer of Americans and accounts for over a sixth of all medical care expenditures.^1,2^ Not all population groups have the same risk for CVD. For instance, Latinos, the largest ethnic minority group in the United States,^3^ have higher rates of CVD behavioral risk factors such as smoking and lack of physical activity than non-Latino whites.^4^ Latinos also have higher rates of CVD clinical risk such as diabetes in older adults^5^ and elevated BMI^6^ as compared with non-Latino whites. Moreover, Mexican-origin Latinos, the largest Latino heritage group, face a unique CVD burden despite having lower rates of CVD risk factors such as hypertension.^7^ Specifically, Mexican-origin Latinos use preventive care services at lower rates,^8^ have lower levels of awareness of hypertensive status, and have lower levels of treatment for hypertension than other Latino heritage groups.^9^ In addition, Mexican-origin Latinos are less likely to receive medication following a myocardial infarction than their non-Latino white peers, increasing their risk for morbidity and mortality.^10^

Improving access to health care may reduce population-level CVD risk.^11^ For example, having health insurance leads to higher rates of CVD diagnoses^12^ and reduces risks of major cardiac events,^13^ suggesting that the disease is more likely identified and controlled among the insured. Lacking health insurance is associated with an increased likelihood of having untreated hypercholesterolemia and hypertension.^14^ Among adults, higher rates of biologically confirmed hypercholesterolemia are seen among the insured than the uninsured.^15^ However, insurance coverage by itself is not enough to confer benefits across all CVD clinical risk factors. For example, adults do not differ in their rates of hypertension, regardless of health insurance status.^15,16^

Insurance status and usual source of care may be linked to CVD by 4 different prevention factors that often vary by race and ethnicity. First, improved health care access increases health care utilization.^17^ However, even when insured, Latinos use fewer health care services than non-Latino whites.^18^ Second, having access to care may facilitate CVD screening, which may prevent or delay the development of the disease and prevent morbidity and mortality. For example, longer term uninsurance is linked to the decreased likelihood of engaging in behaviors that could reduce CVD risk (eg, hypertension and cholesterol screening).^19^ Furthermore, some of the variability in CVD screening is explained by racial and ethnic differences, where ethnic and racial minorities are less likely to be screened compared with non-Latino whites.^20^ Third, being insured or having a usual source of care may increase opportunities for patients to receive preventive care and information from a health care provider targeting CVD risk behaviors. For instance, uninsured obese patients who visited their physicians in the last year are less likely to be advised by their physicians to lose weight than their insured peers.^21^ Moreover, Latinos are less likely than their non-Latino white peers to receive information from health care providers that could reduce their CVD risk.^22^ This is important because research has shown that although individuals receive health information from a range of sources (eg, providers, family, friends, Internet, media), Latinos still have lower levels of health literacy than non-Latino whites,^23^ and generally have less knowledge about CVD.^22,24^ Finally, access to health care may lead to other lifestyle changes (eg, modifying diet, increasing physical activity) that indirectly reduce CVD risk.

Given limited research examining the relation between access to health care and CVD clinical and behavioral risk among Latinos in general, and Mexican-origin Latinos in particular, the present study tested 3 hypotheses: insurance status and usual source of care are associated with the 4 factors linked to CVD prevention (ie, health care utilization, CVD screening, information received from healthcare providers, and lifestyle), insurance status and usual source of care have independent effects, and these relationships depend on the presence or absence of CVD clinical risk/disease (eg, diabetes, heart disease, and high cholesterol). This latter aim tests the assumption that people with underlying CVD risk may receive greater attention from their health care providers and may engage in health-promoting behaviors to curb their CVD risk. In addition, this study will explore what the findings mean for the impact of the Affordable Care Act (ACA) in Latino communities.

## METHODS

All study protocols were approved by the University of California, Los Angeles institutional review board. Written informed consent was obtained from all study participants.

### Setting

The specific context for this study is the neighboring communities of East Los Angeles (East LA) and Boyle Heights. These 2 urban communities in Los Angeles County (LAC), CA, are home to >200,000 people. These communities are disproportionately young, have lower levels of education and income, are majority–Mexican origin, and have a higher proportion of foreign-born residents compared with LAC as a whole.^25–30^ Despite characteristics suggesting socioeconomic disadvantage, these communities are in regions of LAC similar to the rest of the county in terms of rates of hypertension, high cholesterol, and diabetes but have higher rates of obesity.^31^

The high concentrations of Latinos living in East LA and Boyle Heights suggest these communities stand to benefit from the passage of the ACA,^32,33^ which can benefit up to 3.3 million Latinos in the state of California.^34^ However, the ACA will not benefit all Latinos in these communities because the law excludes the undocumented from both the federally funded Medicaid expansion and eligibility for tax credits to purchase insurance through Health Insurance Exchanges.^35^ Unlike other states, California covers recent lawful permanent residents through the state-funded Medicaid program even if they have been in the country <5 years. The communities of East LA and Boyle Heights provide a unique and underexamined context to explore the role of access to care and CVD.

### Design

This cross-sectional study makes use of baseline data collected from a food environment intervention study in East LA and Boyle Heights. The aims of this study, however, are secondary to the primary research and evaluation efforts of the intervention study. A full description of the original study has previously been published.^36^ In short, the intervention aimed to reduce obesity-related chronic disease among Latinos in East LA and Boyle Heights by transforming the food environment. Four corner stores were converted to be healthy food retailers to improve residents’ access to and awareness of fresh and affordable fruits and vegetables in their neighborhoods. Four similar stores served as comparisons.

To assess the effectiveness of the intervention, in-person household surveys were administered in the neighborhoods immediately surrounding each of the intervention and comparison stores. Households were randomly selected, and within the household, the adult (≥18 years) who self-identified as the primary food purchaser and preparer was invited to participate. To ensure consistency in interviewing, in-person interviews were administered using computer-assisted personal interviewing. These interviews took ∼1 hour to complete. Participants had the option of completing interviews in either English or Spanish and received financial incentives for participation. Data were collected between August 2011 and July 2013. To allow adequate opportunity to interview those with varying work schedules and lifestyles, interviews were conducted during daytime, evening, weekday, and weekend.

### Sample

A total of 1381 households were randomly selected for inclusion in the study. Of these, 1199 were screened to be interviewed. Among those who were screened, 90 were ineligible for the study and 72 chose not to participate in the study, leaving 1037 completed interviews. Two surveys were subsequently excluded because they represented duplicate respondents, yielding 1035 completed interviews. The overall response rate was 80%.

Participants were considered for this study only if they responded to questions regarding CVD, which were added midway through baseline data collection. Therefore, 503 participants were eligible to be part of the sample for the present study. Comparability in demographic characteristics, as well as access to care measures, between the full sample and the subsample was assessed. The only statistically significant differences between the groups were in the language spoken at home and insurance status. Specifically, a larger percentage reported speaking both English and Spanish and being insured in the subsample compared with the full sample. The difference in insurance coverage may be attributed to the expansion of low-income insurance coverage in LAC during the data collection period, thus giving those in the subsample more time to benefit from the ACA. Despite these differences, the subsample seems to be a valid representation of the larger sample. Only those individuals with data for all variables of interest were analyzed, yielding an analytic sample of 464.

### Measures

In the current study, there are 4 outcome factors: health care utilization, CVD risk screening, information received from health care professionals, and healthy lifestyle behaviors. Utilization was measured as number of physician visits in last 12 months. Responses were collapsed into no visits versus ≥1 visits. Measures of CVD screening were assessed as time since last blood pressure or cholesterol test. Screening was considered timely based on current American Heart Association screening recommendations for the general adult population (blood pressure in last 2 years and cholesterol in last 5 years).^37,38^ Three items measured whether a health care professional spoke to the participant about his or her weight, eating habits, and exercise (yes vs no). Lifestyle measures represented more healthful behaviors including eating ≥5 servings of fruits and vegetables per day, infrequent (never/sometimes vs often/everyday) consumption of sugar-sweetened beverages (regular soda, fruit drinks, sports drinks, and punch), and exercising for ≥1 hour on ≥5 days in a typical week.

The predictor measure was access to health care. Health care access was measured as insurance status (currently insured vs currently uninsured) and having a usual source of care (yes vs no). The moderator of interest was presence or absence of CVD clinical risk/disease. To assess CVD clinical risk/disease, participants were asked whether a physician had ever told them that they had heart disease, heart failure/congestive heart failure, high blood pressure, high cholesterol, or diabetes/hyperglycemia. An affirmative response to any of these conditions indicated that the respondent had CVD clinical risk/disease. This variable was then multiplied by insurance status and having a usual source of care, to create 2 interaction terms.

Demographic measures included age (in years), sex, years of education, nativity status (US-born vs foreign-born), and language spoken at home (English-only, bilingual, or Spanish-only) and function as control variables.

### Statistical Analysis

IBM Corporation, Armonk, NY, SPSS Statistics version 22.0 was used for statistical analyses. Absolute frequencies, means, and standard deviations were calculated for categorical and continuous variables, as appropriate. Multiple logistic regression analyses were used to estimate the odds ratios for the prevention factors described above for both insurance status and usual source of care, while controlling for demographic measures.^39^ Sequential regression models were fitted, and odds ratios and 95% confidence intervals (CIs) were calculated. The first 2 models analyzed insurance status and usual source of care separately. The next model included both insurance status and usual source in the same model. The last model included variables for CVD clinical risk/disease, the interaction between CVD clinical risk/disease and insurance status, and the interaction between CVD clinical risk/disease and having a usual source of care, to determine whether there are differential effects of insurance status and usual source of care for those who have CVD clinical risk/disease and those who do not. To determine whether logistic regression models fit the data well, model fit was assessed using Hosmer–Lemeshow goodness-of-fit (HL GOF) test. All *P* < 0.05 were considered significant.

## RESULTS

### Missing Data

Out of 503 participants, 92.2% (n = 464) had complete data for all variables of interest and are the focus of analyses in Tables 1 to 4. No respondents were missing information on sex, usual source of care, number of physician visits, blood pressure screening, cholesterol screening, sugar-sweetened beverage consumption, or having CVD clinical risk/disease. One person was missing information on each of the 3 physician recommendation questions and the physical activity question. Two individuals were missing information on insurance and nativity status. Three individuals were missing information on language spoken at home. Fruit and vegetable consumption, education, and age were the questions with the most missing information, with 4, 6, and 24 individuals having missing information on these questions, respectively.

**TABLE 1:**
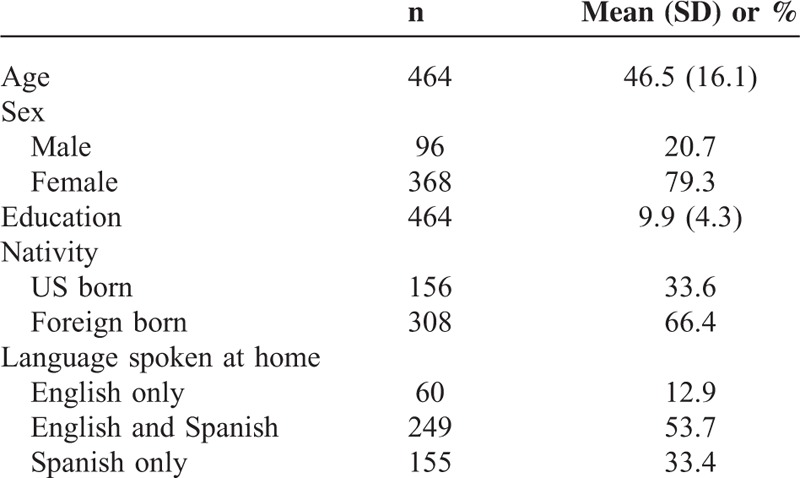
Demographics of the Sample (N = 464)

**TABLE 2:**
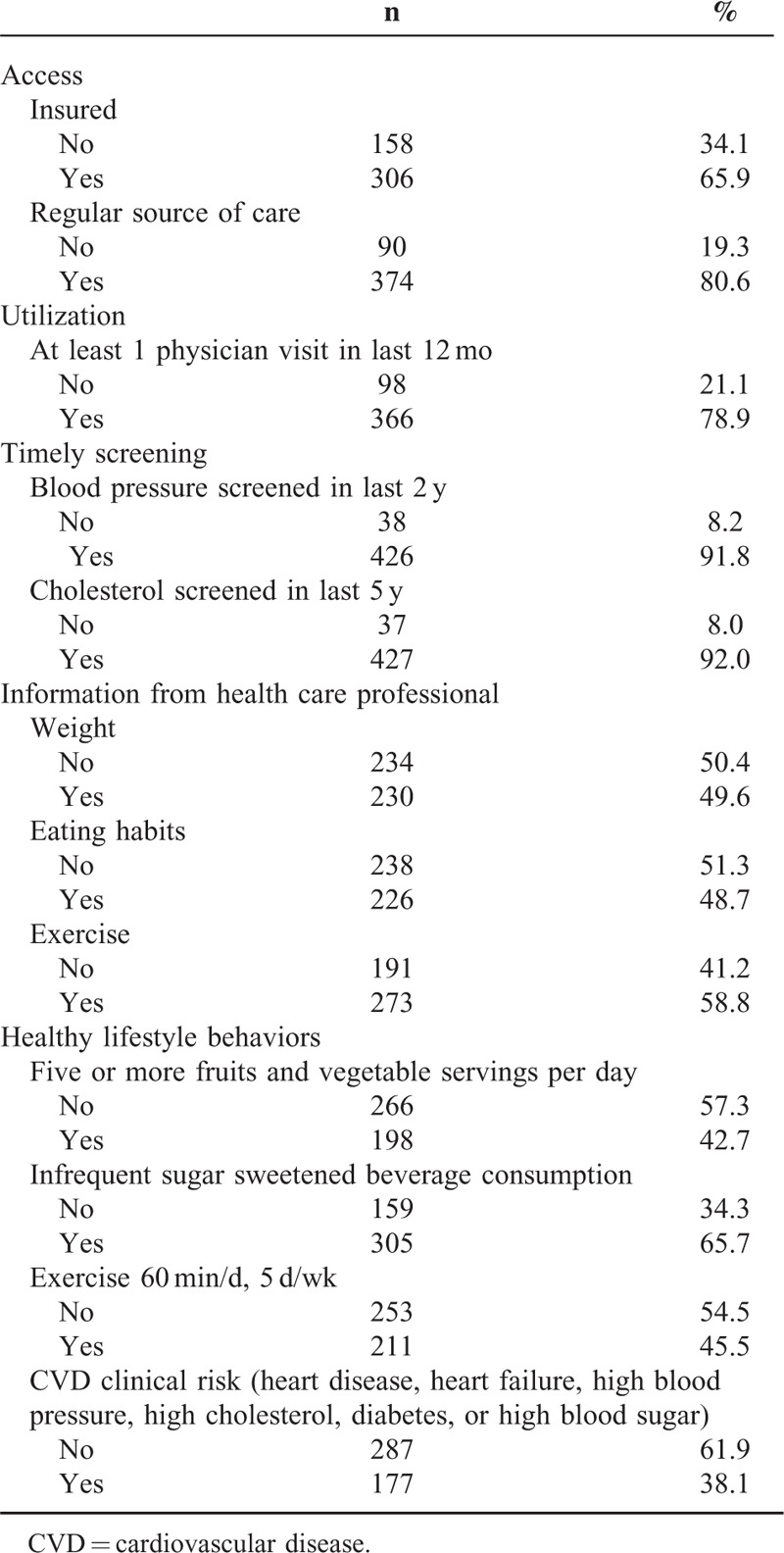
Access, Utilization, Timely Screening, Information Sources, and Lifestyle Behaviors of the Sample (N = 464)

**TABLE 3:**
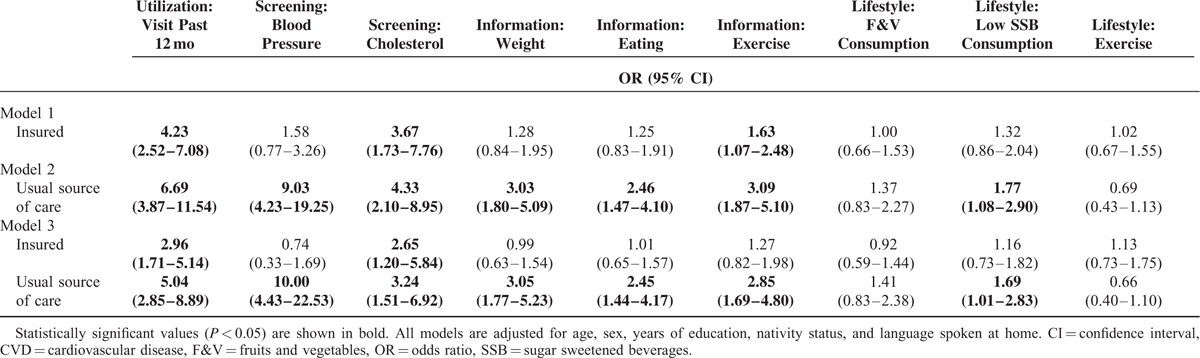
Logistic Regression Models Predicting CVD Prevention Factors

**TABLE 4:**
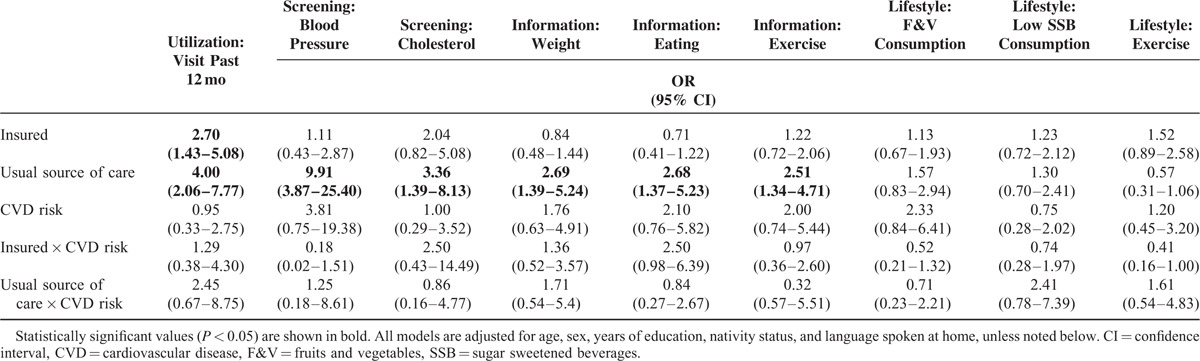
Logistic Regression Models Predicting CVD Prevention Factors With Interactions for Health Conditions

### Descriptive Results

Participants were mostly under the age of 50 years, women, had a high school education or less, were foreign-born, and spoke both English and Spanish at home (Table 1). Most participants were currently insured, had a usual source of care, and had ≥1 physician visits in the last 12 months (Table 2). Participants were relatively healthy with only a third reporting having ever been told they had any type of heart disease, heart failure, high cholesterol, or diabetes/hyperglycemia. Over a quarter of the sample had been told that they had high blood pressure. Nearly all of the participants reported having timely blood pressure and cholesterol screenings. Roughly, half of the sample indicated that a health care professional had spoken to them about their weight and eating habits, and a slightly larger percentage reported having had discussions about exercise. Less than half of the participants indicated they eat ≥5 servings of fruits and vegetables per day or typically exercise for ≥1 hour a day for 5 days a week. Conversely, two-thirds reported infrequent consumption of sugar-sweetened beverages.

### Insurance Status

As Table 3 shows, those with insurance had 4.23 (95% CI 2.52–7.08) times the odds of utilizing health care. Although there were no significant differences in odds of receiving blood pressure screening among the insured and uninsured, there were significantly greater odds of having timely cholesterol screening among those with insurance (OR 3.76, 95% CI 1.73–7.76). Being insured was associated with 1.63 times the odds (95% CI 1.07–2.48) of talking about exercise with a health care professional. However, being insured did not increase the odds of having discussions about weight or eating. In addition, being insured was not associated with greater odds of engaging in healthy behaviors. When usual source of care was entered into the model, the association between insurance status and talking about exercise with a health care professional became nonsignificant. All of the models fit the data reasonably well (HL GOF *P* > 0.05).

### Usual Source of Care

Participants who reported having a regular source of care had 6.69 times the odds (95% CI 3.87–11.54) of having ≥1 physician visit in the last 12 months. In addition, they had significantly greater odds of timely screening for both blood pressure (OR 9.03, 95% CI 4.24–19.25) and cholesterol (OR 4.33, 95% CI 2.10–8.95). Further, having a regular source of care was associated with increased odds of discussing weight (OR 3.03, 95% CI 1.80–5.09), eating (OR 2.46, 95% CI 1.47–4.10), and exercise (OR 3.09, 95% CI 1.87–5.10) with a health care professional. Those with a regular source of care also had increased odds of drinking sugar-sweetened beverages less frequently (OR 1.77, 95% CI 1.08–2.90). When insurance status was entered into the model, all of the previously significant associations remained significant. All models fit the data reasonably well (HL GOF *P* > 0.05).

### Insurance Status and Usual Source of Care Moderated by CVD Clinical Risk/Disease

Table 4 shows that none of the relationships between insurance status or a usual source of care and CVD prevention factors depended on the presence or the absence of CVD clinical risk/disease. Among those with no CVD clinical risk/disease, being currently insured was associated with increased odds of having ≥1 physician visit in the last 12 months (OR 2.70, 95% CI 1.43–5.08). In addition, having a usual source of care was associated with increased odds having ≥1 physician visit in the last 12 months (OR 4.00, 95% CI 2.06–7.77), having a timely screening for blood pressure (OR 9.91, 95% CI 3.87–25.40), and having timely cholesterol screening (OR 3.36, 95% CI 1.39–8.13). Those with no CVD clinical risk/disease and a usual source of care had increased odds of discussing weight (OR 2.69, 95% CI 1.39–5.24), eating (OR 2.68, 95% CI 1.37–5.23), and exercise (OR 2.51, 95% CI 1.34–4.71) with a health care professional. All of the models fit the data reasonably well (HL GOF *P* > 0.05).

In separate analyses, among those with CVD risk, being currently insured was associated with increased odds of having ≥1 physician visit in the last 12 months (OR 3.47, 95% CI 1.21–9.96) and having timely cholesterol screening (OR 5.11, 95% CI 1.11–23.56). Among those with CVD risk, having a usual source of care was associated with increased odds of having ≥1 physician visit in the last 12 months (OR 9.81, 95% CI 3.27–29.43), having timely cholesterol screening (OR 12.40, 95% CI 2.32–66.32), and drinking sugar-sweetened beverages less frequently (OR 3.12, 95% CI 1.22–8.00). Those with CVD clinical risk/disease and a usual source of care had increased odds of discussing weight (OR 4.59, 95% CI 1.77–11.93) and exercise (OR 4.46, 95% CI 1.71–11.57) with a health care professional. All of the models fit the data reasonably well (HL GOF *P* > 0.05).

## DISCUSSION

To the authors’ knowledge, few studies have examined the relation between access to care and CVD prevention among Latinos. In this study of primarily Mexican-origin Latinos in 2 communities, having a usual source of care was a robust predictor of the 4 prevention factors (ie, health care utilization, CVD screening, information received from healthcare providers, and lifestyle). Insurance status was independently associated with only 2 of the 4 prevention factors. Contrary to what was originally hypothesized, these findings suggest that insurance coverage alone has a limited affect on CVD prevention, when compared with having a usual source of care. Also, because having a usual source of care is far more common than being insured among the study population, our findings suggest that individuals may be relying on safety net clinics for care or utilizing complementary or alternative medicine.

In addition, contrary to our initial hypothesis that those with CVD clinical risk/disease would have differential rates of utilization, screening, receiving information, and healthier lifestyle behaviors than those without CVD; our findings show that these prevention factors did not depend on CVD clinical risk/disease. This is counter to the notion that high-risk individuals should be receiving more targeted care from their health care providers. Overall, this suggests that delivery of care to Latinos with CVD risk can be improved.

### Implications for the ACA

The findings from this study have important implications for the implementation of the ACA in Latino communities. Given the high levels of uninsurance in East LA and Boyle Heights, efforts to increase access to insurance have the potential to improve CVD prevention. However, the study shows that focusing only on increasing insurance coverage, and not on improving access to usual sources of care, may have a limited impact on increasing access to screening, behavioral advice, or translate into more beneficial health behaviors. This is unsurprising considering insurance coverage is only 1 component of the access-to-care-puzzle.^40,41^ In addition, recent work has shown that although the ACA has increased insurance coverage across racial/ethnic groups, it has not decreased the racial/ethnic disparities in insurance coverage^42^ and will likely not decrease disparities in CVD health. The ACA, however, provides concrete coverage benefits to those who already have CVD because the policy prohibits the denial of coverage or higher insurance premiums for those with preexisting conditions.^43^

The passage of the ACA may also significantly impact where Latino patients receive care.^32^ In Latino neighborhoods such as East LA and Boyle Heights, there are a number of low-cost, federally qualified health centers and other community clinic providers that serve these communities. These safety net sources of medical care may see their patient mix change, and the number of people they serve dwindle, as ACA provisions are implemented. Specifically, as US citizens gain insurance coverage, they may move away from safety net clinics, whereas the undocumented, who are excluded from the ACA's expansion of insurance coverage, will not have this option. As such, these clinics will need to adjust their services to meet the needs of an increasingly younger, foreign-born, and non-English speaking population.

Overall, insurance expansion provisions in the ACA cannot be expected to provide a comprehensive path to lowering CVD population risk because having insurance alone does not positively improve all CVD risk factors. The ACA's ability to improve CVD health and reduce CVD health disparities may be limited among populations where sizable groups are excluded from insurance expansion (ie, the undocumented). The ACA has the potential to improve CVD health in ways that go beyond the provision of health insurance. For example, the ACA provides grants to small businesses to implement workplace health programs that may help prevent CVD and rehabilitate those who already have CVD.^44^ In addition, the passage of the ACA created the Prevention and Public Health Fund, which aims to provide the opportunity to prevent disease, both inside and outside the access-to-care context.^45^ As such, workplace health program and Prevention and Public Health Fund are just 2 examples of funding mechanisms under the ACA that are designed to support prevention efforts outside the medical care setting. Moreover, these provisions have the potential to improve population health and reduce CVD for those with and without access to insurance.

### Limitations

Although this study's findings help to clarify the relation between access to care and CVD risk among those who live in Latino-concentrated communities, limitations must be considered. First, the sample is largely young and women, which are groups with lower CVD risk. These individuals may not be typical targets for physician interventions for CVD. This may, in turn, bias findings toward the null. Second, because the sample is mostly women and utilized unique selection criteria for study participants, the generalization of the findings might be hampered. Third, because the present study relies on self-reported measurements, reporting bias cannot be ruled out. Despite these limitations, the current study provides a broad examination of the role of access to care and modifiable CVD risk factors among Latinos.

## CONCLUSIONS

This study showed potential means by which access to care can influence CVD prevention. With the ACA's insurance expansion currently underway, the study highlights the potential benefit this can provide, in terms of cardiovascular health to Latino communities. Access to insurance is a critically important step but alone will be insufficient to reduce CVD rates. As such, efforts should be made to strengthen the relationship between providers and patients, by using population health approaches that consider patient characteristics, health promotion, and medical care.^46^ This begins with health care practices providing more comprehensive services and having a whole person orientation. Moreover, efforts to reduce CVD need to go beyond traditional medical encounters. Development and implementation of culturally relevant health promotion campaigns and interventions that focus on improving health literacy, increasing knowledge, and promoting health behaviors are essential to improving population health. Although these additional efforts are addressed by some components of the ACA, they receive considerably less funding and attention than insurance expansion.
